# High prevalence of vascular calcification in young stroke patients

**DOI:** 10.1016/j.ajpc.2026.101647

**Published:** 2026-04-20

**Authors:** Sarina Chadha, Gourisree Dharmavaram, Brielle Martinez, Ayaan Mahmud, Dahlia Adler, Raphael Cuomo, Harpreet S. Bhatia, Mattheus Ramsis, Michael J. Wilkinson, Brett C. Meyer, Pam R. Taub

**Affiliations:** a University of California, San Diego, School of Medicine, 9500 Gilman Dr, La Jolla, CA 92093, United States; b University of California, San Diego, Department of Medicine, Division of Cardiology, 9434 Medical Center Drive, San Diego 92037, USA; c University of California, San Diego, Department of Neurosciences, Division of Stroke, 9434 Medical Center Drive, San Diego 92037, USA

## 1 Introduction

Stroke incidence among younger adults has increased substantially over the past two decades, yet the contribution of subclinical vascular disease in this population remains incompletely characterized [1,2]. Vascular calcification reflects cumulative vascular injury and is associated with increased risk of cardiovascular events [4,5]. However, the prevalence and distribution of calcification across vascular beds in young stroke patients have not been comprehensively evaluated. In our single-center cohort study of adults ≤ 60 years presenting with stroke, vascular calcification was present in nearly two-thirds of patients, highlighting an underrecognized substantial burden of systemic vascular disease in a population often considered at low cardiovascular risk.

## 2 Methods and study population

We conducted a retrospective cohort study of adults ≤ 60 years admitted with ischemic stroke, intracerebral hemorrhage (ICH), or subarachnoid hemorrhage (SAH) at University of California San Diego (UCSD) Health between November 2, 2021 and April 28, 2025. Patients were identified from the Get With the Guidelines-Stroke registry and linked electronic medical records. Patients older than 60 years, those with stroke mimics, or with incomplete records were excluded. For individuals with multiple admissions, the first qualifying event was included. The UCSD Institutional Review Board approved the study with waiver of informed consent.

Demographic and clinical data were obtained from the electronic medical record. Stroke etiology was classified using Trial of ORG 10,172 in Acute Stroke Treatment (TOAST) criteria. Neurovascular and systemic imaging performed within six months before or after the index stroke was reviewed. Imaging included CT/CTA of the head and neck, chest or abdomen, carotid ultrasound or MR angiography, and transthoracic echocardiograms. Vascular calcification (VC) was defined as documented calcification in the official radiology or echocardiography report and categorized as present or absent for each vascular bed, including coronary, carotid, intracranial, thoracic aorta, aortic valve, and mitral annular locations. Imaging reports were reviewed retrospectively using a standardized abstraction form with adjudication by the principal investigator when necessary.

### 2.1 Study outcomes

The primary outcome was the presence of calcification in any vascular bed. Secondary outcomes included the distribution of VC across beds and associations with baseline comorbidities.

### 2.2 Statistical analyses

Continuous variables were summarized as median with interquartile range and categorical variables as counts and percentages. Comparisons between patients with and without calcification were performed using Mann-Whitney U tests for continuous variables and chi-square tests for categorical variables. Multivariable logistic regression evaluated the independent associations between age, sex, hypertension, diabetes, and serum creatinine and vascular calcification.

## 3 Results

**Fig. 1:**
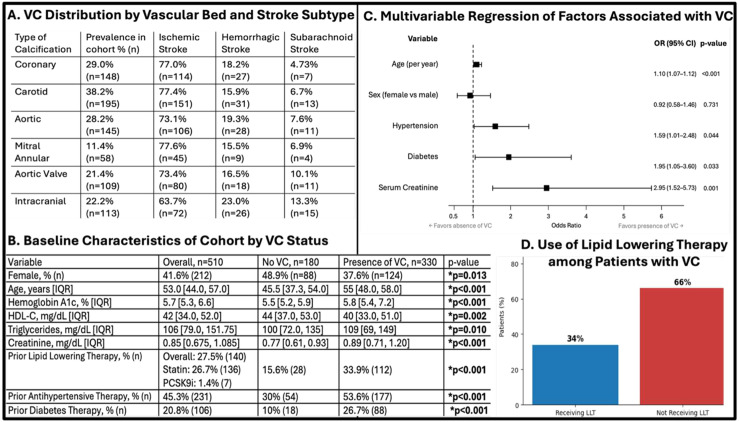
Systemic Vascular Calcification in Young Adults with Stroke. Panel A. Distribution of VC across Vascular Beds in the Study Cohort,Stratified by Stroke Subtype. Panel B. Baseline Clinical and Cardiometabolic Characteristics of the Study Cohort. Panel C. Multivariable Logistic Regression Analysis of Factors associated with VC. Panel D. Use of Lipid Lowering Therapy among Patients with VC. Abbreviations: VC = vascular calcification, OR = odds ratio, CI = confidence interval.

Among 510 adults ≤ 60 years with stroke (median age 53 years [IQR 44–57],42% women), vascular calcification in at least one vascular bed was identified in 330 patients (65%). Significant baseline characteristics are summarized in Fig. 1B. Patients with calcification were older than those without calcification (median age 55 vs. 45.5 years, p < 0.001) and had higher hemoglobin A1c levels and lower HDL-C (Fig. 1B). LDL-C did not differ significantly between groups (median 90 mg/dL in both groups). Lipid lowering therapy (LLT) prior to the index event was used in 34% of patients with documented vascular calcification (Fig. 1D).

Calcification was observed across multiple vascular beds (Fig. 1A). The most frequently involved territories were the carotid arteries (38%), coronary arteries (29%), and thoracic aorta (28%). Intracranial arterial calcification was present in 22% of patients, while valvular calcification was also common, including aortic valve calcification in 21% and mitral annular in 11%. Calcification was most prevalent among patients with ischemic stroke but also observed in patients presenting with ICH and SAH (Fig. 1A).

Among patients with ischemic stroke, 41% were classified as cryptogenic according to TOAST criteria. In this subgroup, patent foramen ovale was present in 61% of patients and vascular calcification was present in 66% of patients. Coronary calcification was the most common (37%) followed by carotid calcification (27%), aortic valve calcification (25%), intracranial arterial calcification (20%), and mitral annular calcification (16%).

In multivariable regression analysis, older age was strongly associated with VC (odds ratio [OR] 1.10, 95% CI 1.07–1.12, p < 0.001). Hypertension (OR 1.59, 95% CI 1.01–2.48, p = 0.044), diabetes mellitus (OR 1.95, 95% CI 1.05–3.60, p = 0.033), and serum creatinine (OR 2.95, 95% CI 1.52–5.73, p = 0.001) were independently associated with calcification, whereas sex was not (Fig. 1C).

## 4 Discussion

In this cohort of adults ≤ 60 years with stroke, vascular calcification was highly prevalent and frequently involved multiple vascular territories. Although calcification was most common among patients with ischemic stroke, it was also observed among patients with hemorrhagic stroke subtypes, suggesting that vascular changes are not limited to a single stroke subtype.

VC has traditionally been interpreted as a marker of cumulative atherosclerotic burden and vascular injury rather than a direct embolic source [[4], [5], [6]]. Mechanistically, calcification reflects processes including vascular smooth muscle cell osteogenic transformation, arterial stiffening, endothelial dysfunction, and chronic metabolic stress [6]. These changes may impair cerebral vascular compliance and distal perfusion while also increasing susceptibility to vessel-wall injury [6].

The high prevalence of calcification observed in patients classified as having cryptogenic stroke highlights the complexity of stroke mechanism attribution in younger adults [3]. Although many of these patients underwent evaluation for paradoxical embolism, including identification of a patent foramen ovale, the frequent coexistence of systemic VC suggests that unrecognized vascular disease may contribute to cerebrovascular risk in a subset of patients currently classified as cryptogenic. These findings underscore the importance of considering systemic vascular pathology when evaluating stroke etiology in younger individuals.

Multivariable analyses further support the concept of vascular calcification as a marker of systemic vascular disease. While age remained the strongest determinant of calcification, hypertension, diabetes, and renal dysfunction were independently associated with its presence. These findings reinforce VC as a marker of systemic vascular disease beyond aging alone.

Our findings are consistent with prior population-based studies demonstrating that calcification frequently develops before overt cardiovascular disease [4,5]. Importantly, lipid-lowering therapy was only used in approximately one-third of patients with documented vascular calcification (Fig. 1D). Recent 2026 ACC/AHA/Multisociety Dyslipidemia Guidelines emphasize the clinical importance of subclinical atherosclerosis detected on imaging, recommending consideration of lipid-lowering therapy in patients with incidental CAC identified on noncardiac CT scans [7]. Although these recommendations primarily focus on CAC, the low use of LLT observed in our cohort, despite a high prevalence of systemic VC across multiple vascular beds, highlights a potential gap between detection of subclinical vascular disease and implementation of preventive strategies. Recognition of incidental calcification on routine imaging may therefore represent a practical opportunity to improve cardiovascular risk assessment and initiate preventive therapy.

This study has several limitations. It was conducted at a single center and relied on retrospective review of clinically acquired imaging. Calcification was identified qualitatively from imaging reports rather than quantitatively using standardized scoring systems. Imaging was clinically acquired rather than protocolized, and neurovascular imaging modalities were available in most patients (93.9%), whereas coronary imaging was performed selectively and less frequently (8.8%). Many of these factors may have influenced the relative under-estimation of calcification across vascular beds. Finally, the absence of a non-stroke control group limits causal inference regarding the relationship between calcification and stroke risk.

## 5 Conclusion

In adults ≤ 60 years presenting with stroke, vascular calcification was frequently observed across multiple vascular beds but remained significantly undertreated. These findings highlight vascular calcification as an underrecognized marker of systemic vascular disease and support further study of calcification-guided preventive strategies in younger stroke populations.

## Sources of support

Research Funded by UC San Diego BEACON. The funder had no role in the design and conduct of the study; collection, management, analysis or interpretation of the data; preparation, review, or approval of the manuscript; or decision to submit the manuscript for publication.

## Ethical review statement

This manuscript is original, has not been published previously, and is not under consideration elsewhere. If accepted, it will not be published elsewhere without written permission from the journal.

## CRediT authorship contribution statement

**Sarina Chadha:** Writing – review & editing, Writing – original draft, Visualization, Supervision, Software, Project administration, Methodology, Investigation, Formal analysis, Data curation, Conceptualization. **Gourisree Dharmavaram:** Writing – review & editing, Writing – original draft, Visualization, Supervision, Software, Project administration, Methodology, Investigation, Formal analysis, Data curation, Conceptualization. **Brielle Martinez:** Writing – review & editing, Writing – original draft, Visualization, Supervision, Methodology, Investigation, Data curation. **Ayaan Mahmud:** Writing – review & editing, Writing – original draft, Visualization, Supervision, Methodology, Investigation, Data curation. **Dahlia Adler:** Writing – review & editing, Writing – original draft, Visualization, Supervision, Methodology, Investigation, Data curation. **Raphael Cuomo:** Writing – review & editing, Writing – original draft, Visualization, Supervision, Software, Project administration, Methodology, Investigation, Formal analysis, Data curation. **Harpreet S. Bhatia:** Writing – review & editing, Writing – original draft, Visualization, Supervision, Software, Resources, Project administration, Methodology, Investigation, Funding acquisition, Formal analysis, Conceptualization. **Mattheus Ramsis:** Writing – review & editing, Writing – original draft, Visualization, Supervision, Software, Resources, Project administration, Methodology, Investigation, Funding acquisition, Formal analysis, Conceptualization. **Michael J. Wilkinson:** Writing – review & editing, Writing – original draft, Visualization, Supervision, Resources, Project administration, Methodology, Investigation, Funding acquisition, Formal analysis, Conceptualization. **Brett C. Meyer:** Writing – review & editing, Writing – original draft, Visualization, Supervision, Software, Resources, Project administration, Methodology, Investigation, Funding acquisition, Formal analysis, Data curation, Conceptualization. **Pam R. Taub:** Writing – review & editing, Writing – original draft, Visualization, Supervision, Software, Resources, Project administration, Methodology, Investigation, Funding acquisition, Formal analysis, Data curation, Conceptualization.

## Declaration of competing interest

Dr. Bhatia– consulting/advisory board for Abbott, Arrowhead, Bayer, Kaneka, New Amsterdam, Novartis; funding National Institutes of Health, Grant 1K08HL166962 Dr. Meyer– consultant for Sevaro Health. CMO InSightVue
